# Rapid and Facile Preparation of Giant Vesicles by the Droplet Transfer Method for Artificial Cell Construction

**DOI:** 10.3389/fbioe.2022.873854

**Published:** 2022-04-07

**Authors:** Yasuhiro Shimane, Yutetsu Kuruma

**Affiliations:** ^1^ Institute for Extra-cutting-edge Science and Technology Avant-garde Research (X-star), Japan Agency for Marine-Earth Science and Technology (JAMSTEC), Yokosuka, Japan; ^2^ Research Institute of Industrial Technology, Toyo University, Saitama, Japan; ^3^ PRESTO, Japan Science and Technology Agency (JST), Saitama, Japan; ^4^ Graduate School of Nanobioscience, Yokohama City University, Yokohama, Japan

**Keywords:** giant vesicle, artificial cells, protocells, droplet transfer, cell-free synthetic biology, cell-free protein synthesis, liposome, lyophilization

## Abstract

Giant vesicles have been widely used for the bottom-up construction of artificial (or synthetic) cells and the physicochemical analysis of lipid membranes. Although methods for the formation of giant vesicles and the encapsulation of molecules within them have been established, a standardized protocol has not been shared among researchers including non-experts. Here we proposed a rapid and facile protocol that allows the formation of giant vesicles within 30 min. The quality of the giant vesicles encapsulating a cell-free protein expression system was comparable to that of the ones formed using a conventional method, in terms of the synthesis of both soluble and membrane proteins. We also performed protein synthesis in artificial cells using a lyophilized cell-free mixture and showed an equivalent level of protein synthesis. Our method could become a standard method for giant vesicle formation suited for artificial cell research.

## Introduction

The droplet transfer method, which is also known as the inverted emulsion method, has been widely used for the formation of giant vesicles (GVs) with a diameter of tens of micrometers (Pautot et al., 2003; Walde et al., 2010). GVs have been applied as a model cell membrane for the investigation of the physical properties of lipid membranes in the field of soft-matter physics (Jimbo et al., 2016; Oda et al., 2020; Lowe et al., 2022), and for the construction of artificial cells in the field of synthetic biology (Lu et al., 2021). Although several methods have been developed to form GVs, such as electro formation (Angelova and Dimitrov, 1986) or film hydration (Reeves and Dowben, 1969; Tsumoto et al., 2009), the droplet transfer method (Moga et al., 2019) has advantages in the generation of single lamellar membrane vesicles and its application to biochemical experiments requiring physiological conditions. The principle of this method is as follows: the water-in-oil (W/O) droplets, which are stabilized by a monolayer of phospholipids, pass through another monolayer formed at the interface of a lipid-oil layer and an aqueous layer; as a consequence, lipid bilayer vesicles are formed in the aqueous solution (Noireaux and Libchaber, 2004).

Although GVs are highly versatile materials, it is not always easy to obtain quality GVs with high reproducibility, which often fails even if following the same experimental steps. Moreover, beginners who have no experience in preparing GVs may be more likely to have an experimental failure. This tendency increases as the composition of the internal solution become more complex. To reduce these difficulties, some tailor-made devices have been created and applied in the droplet transfer method (Morita et al., 2018; Bashirzadeh et al., 2021). In addition to the droplet transfer method, more advanced methods using microfluidic devices have recently been developed and will become the main technology in this field (Robinson, 2019; Kamiya, 2020). However, designing the devices, creating the template mold, and the relatively high cost for the system set up hesitate us to choose this method. Therefore, establishing a concise protocol that allows the easy and rapid preparation of GVs with high reproducibility is important to reduce experimental errors.

In this report, we present a handy protocol that allows the completion of all experimental steps, from the lipid-oil preparation to the GV formation, within 30 min without the use of specific devices. The reproducibility of the method is high and a sufficient population of GVs can be produced. We show that we have successfully prepared artificial cells by this method using a cell-free protein expression system (cell-free system) that has been lyophilized and rehydrated. Because the technique of GV formation is the basis of artificial cell experiments, we aim to standardize our method for the development of artificial (synthetic) cell research.

### Overview of the Experimental Approach Used for GV Formation

**FIGURE 1:**
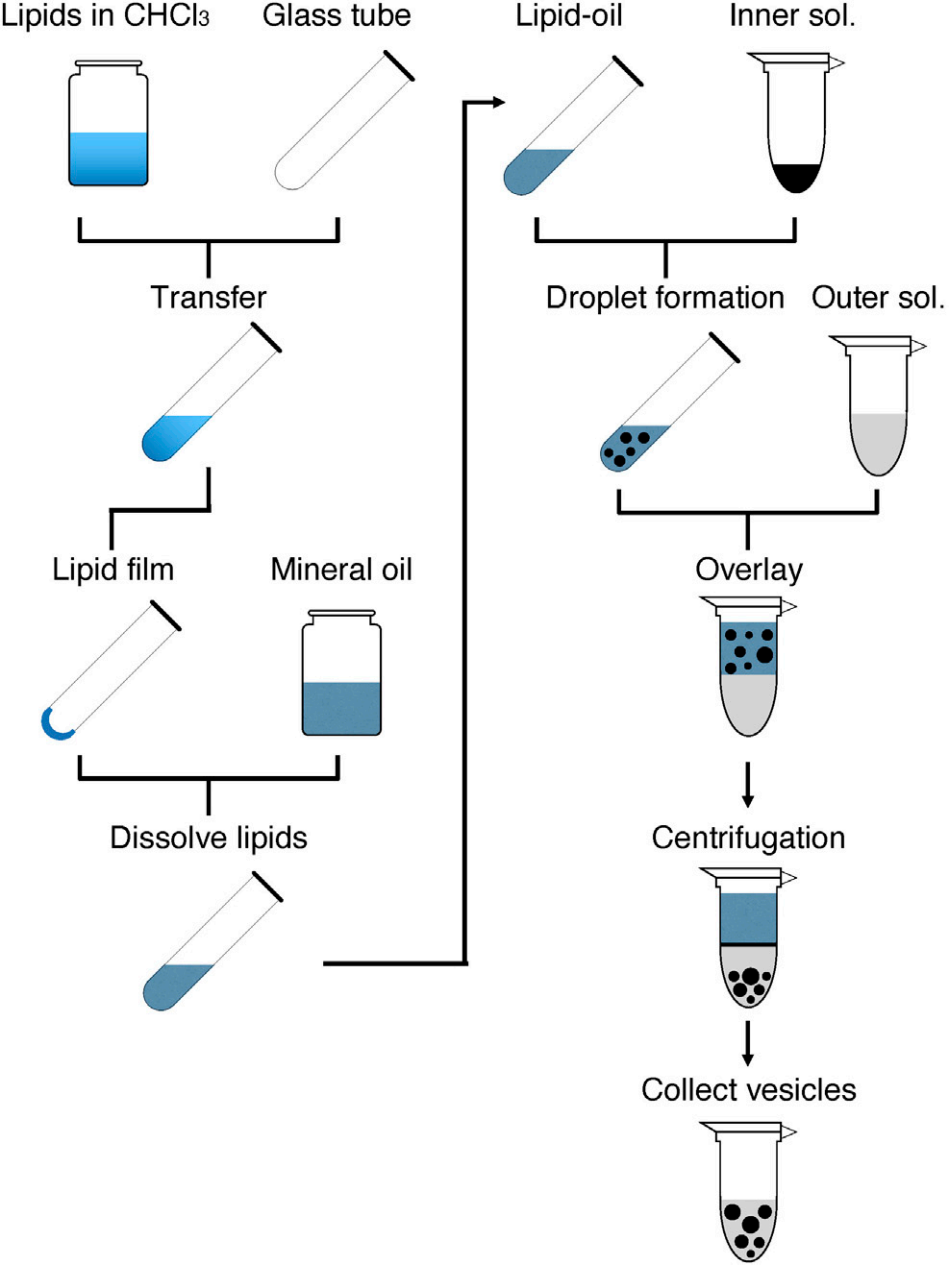
Overview of the rapid and facile preparation of giant vesicles. The left half shows the steps for the preparation of lipid-oil mixture, and the right half shows the steps for the formation of GVs. The details are described in the Stepwise Procedures.

As shown in Figure 1, GV formation is initiated by preparing a lipid-oil mixture containing the desired lipid composition. The prepared lipid-oil mixture is used to form W/O droplets by mixing with the inner solution, which is composed of the desired reaction mixture, e.g., a cell-free system. The prepared droplets are layered on the outer solution. Giving a force by centrifugation induces the passage of the droplets through the interface between the oil and aqueous layers, thus forming lipid bilayer GVs in the aqueous solution. The formed GVs are collected and used for a subsequent experiment. If needed, the GVs can be washed with the outer solutions to remove the components that leaked from the broken droplets.

### Lipid Composition of GV Membranes

Although the type of lipids used for GV formation depends on the purpose of each experiment, 1-palmitoyl-2-oleoyl-sn-glycero-3-phosphocholine (POPC) or 1,2-dioleoyl-sn-glycero-3-phosphocholine (DOPC) is generally used as a basic lipid, because of its high stability. When preparing GV using several types of lipids, the lipids dissolved in an organic solvent such as chloroform at the defined concentration are mixed to obtain the desired composition. When synthesizing membrane proteins inside GVs, an acidic phospholipid such as phosphatidylglycerol (PG) or phosphatidylserine (PS) is added to the lipid composition—for example, 10–30 mol% acidic phospholipids are used—because the negative charge on the membrane surface is important for maintaining the correct structure of membrane protein and affects to its function (Pöyry and Vattulainen, 2016). The charge is also important for the localization of peripheral membrane proteins onto the membrane surface (Furusato et al., 2018). The use of 30 mol% cholesterol makes the membrane rigid (Gracià et al., 2010; Chakraborty et al., 2020). However, it should be noted that a certain amount of cholesterol inhibits the spontaneous membrane insertion when integral membrane proteins were synthesized inside GVs or outside of liposomes (Nakamura et al., 2018; Berhanu et al., 2019). Besides cholesterol, a physiological concentration of diacylglycerol also inhibits the spontaneous insertion (Nakamura et al., 2018). Fluorescent lipids or hydrophobic dyes, such as rhodamine-phosphatidylethanolamine (PE), NBD-PE, or Nile Red, are used for labeling GV membranes. Moreover, polyethylene glycol (PEG)-lipids (2–5 KDa in size) are used to avoid the adhesion of GVs. Chemically modified unnatural lipids are also used for experimental purposes (Kurihara et al., 2011; Bhattacharya et al., 2019). Conversely, natural lipids extracted from the membranes of various organisms, such as soybean or egg yolk, are not suitable for the formation of stable GV by this method, whereas they are applicable for the electroformation method (Méléard et al., 2009) or for preparation of small-size liposomes (<200 nm in diameter). Examples of lipid compositions reported in recent artificial cell studies are shown in Table 1.

**TABLE 1:** Lipid composition of GV used for artificial cell experiments.

Type of synthesized or encapsulated proteins	Lipid composition (mol%)	References
Integral membrane proteins	DOPC 50%, DOPE 36%, DOPG 12%, CL (18:1) 2%, DSPE-PEG-biotin 1 mass%, DHPE-Texas Red 0.5 mass%. Or, DOPC 75%, DOPG 25%, DSPE-PEG-biotin 1 mass%, DHPE-Texas Red 0.5 mass%	Blanken et al. (2020)
Soluble and integral membrane proteins	POPC and cholesterol with or without 1% DSPE-PEG(2000) biotin	Toparlak et al. (2020)
Soluble protein	DOPC and a chemically modified lysophospholipid	Bhattacharya et al. (2019)
Soluble protein	POPC 40%, POPE 20%, POPG 20%, cholesterol 20%, NBD-PE 0.25%	Lee et al. (2018)
Soluble and integral membrane proteins	POPC 57.5%, cholesterol 40%, PEG2000PE^a^ 0.25%	Berhanu et al. (2019)
Integral and peripheral membrane proteins	POPC 80%, POPG 20%, rhodamine-DOPE 0.5%	Furusato et al. (2018)

a 1,2-distearoyl-*sn*-glycero-3-phosphoethanolamine-*N*-[biotinyl(polyethyleneglycol)-2000].

### Inner Solution

The inner solution corresponds to the internal aqueous phase of GVs. Regarding the inner solution, any component can be encapsulated in GVs, except hydrophobic molecules and detergents. For example, by adding purified proteins into the inner solution, various enzymatic reactions or structural formations in the GVs can be generated, e.g., polymerase chain reaction (Oberholzer et al., 1995), transcription (Altamura et al., 2021), actin polymerization (Lee et al., 2018; Litschel et al., 2021), Min oscillation (Litschel et al., 2018; Yoshida et al., 2019), etc. In more advanced applications, the encapsulation of a cell-free system reconstituted with purified multi-components involved in transcription and translation (Shimizu et al., 2001) or a cell extract from a certain organism such as *Escherichia coli* (Noireaux and Liu, 2020) allows the synthesis of desired proteins from the genes of interest. This technology has an advantage, particularly for the synthesis of membrane proteins (Furusato et al., 2018; Berhanu et al., 2019). In general, the purification of membrane proteins requires the use of a detergent that dissolves the cell membrane to isolate the proteins. The purified membrane protein sample contains a detergent, therefore it cannot be encapsulated inside GVs, although there are some exceptions (Yanagisawa et al., 2011; Altamura et al., 2017). To solve this problem, synthesizing membrane proteins inside GVs is a rational approach and allows the expression of biochemical functions on the GV membranes. This approach is depending on the phenomenon of spontaneous membrane insertion of nascent membrane proteins (Nishiyama et al., 2006). It should be noted that, when a certain amount of cholesterol or diacylglycerol is included in the composition of the GV membrane, this spontaneous insertion does not occur. The limitation of this approach is that not all membrane proteins can be integrated into the lipid membrane in a native form (Berhanu et al., 2019). For example, if a membrane protein has a large hydrophilic domain at the outside of the GV membrane (the opposite side of the translating ribosome), the spontaneous insertion with the correct membrane orientation of the protein does not occur.

In addition to purified proteins and cell-free systems, small-sized liposomes with a diameter of <200 nm can also be encapsulated, presenting the vesicle-in-vesicle structure (Lee et al., 2018; Berhanu et al., 2019; Altamura et al., 2021). This technique can mimic intracellular organelles. Interestingly, by coupling with a cell-free system that synthesizes a membrane protein, it is possible to localize the synthesized membrane proteins onto the liposome membrane (Berhanu et al., 2019). The membrane localization of the protein can be oriented using cholesterol (Nakamura et al., 2018), i.e., when cholesterol is used in the GV membrane but not in the liposome membrane, a large part of the synthesized membrane proteins are localized to the internal liposomes. The encapsulated liposome organelle is also useful for generating a proton gradient between the GV lumen and the liposome inside (Berhanu et al., 2019).

To mimic intracellular molecular crowding, Ficoll PM70 (Furusato et al., 2018; Berhanu et al., 2019) or bovine serum albumin (Fujiwara and Yanagisawa, 2014) is often encapsulated within GVs. This molecular crowding effect is essential for various types of molecular assembly and, especially, for the membrane localization of peripheral membrane proteins in association with a negative charge on the membrane surface.

In any case, the droplet transfer method generally uses sucrose in the inner solution to make it heavier than the glucose-containing outer solution, while maintaining equal osmotic pressure. In some experiments, sucrose may have a side effect on the internal reaction. In such a case, OptiPrep, a density gradient medium, is often used instead of sucrose (Van de Cauter et al., 2021).

An example of the inner solution used for the encapsulation of a commercial cell-free kit (PURE system) in GVs is shown in Table 2.

**TABLE 2:** Example of the inner solution of artificial cells using the PURE system (PURE*frex*2.0).

Reagent	Volume (µL)
Sol. I (Buffer, etc.)	10
Sol. II (Enzyme mix)	1
Sol. III (Ribosomes)	2
2 M Sucrose	2
DNA (plasmid or linear)	X (1–5 nM f.c.)
Water	5-X
Total	20

### Outer Solution

The outer solution should be made when preparing the inner solution or in advance. The composition of the outer solution should be same or very similar to that of the inner solution, except reactive molecules and genes, i.e., enzymes and DNAs. The osmolality of the outer solution should be adjusted by changing the concentration of glucose, or alternative material, to be the same as the inner osmotic pressure. For the use of cell-free systems, the basic buffer composition and amino acids should be maintained, whereas a tRNA mixture and NTPs are removed. Note that the outer solution should be prepared from a double-concentrated solution, as the final solution can be diluted with the glucose solution (see an example in Table 3). The prepared outer solution can also be used for washing GVs after their formation.

**TABLE 3:** Example of the outer solution of artificial cell encapsulating the PURE system.

Component	2× preparation	1× final conc
HEPES-KOH (pH 7.6)	40 mM	20 mM
Potassium glutamate	360 mM	180 mM
MgOAc	28 mM	14 mM
Spermidine	4 mM	2 mM
10-Formyl-tetrahydrofolate	20 μg/ml	10 μg/ml
Dithiothreitol	4 mM	2 mM
18-amino-acid mixture (w/o cysteine and tyrosine)	1 mM	0.5 mM
Cysteine	1 mM	0.5 mM
Tyrosine	1 mM	0.5 mM
Creatine phosphate	40 mM	20 mM

### Formation of GVs

The prepared W/O droplets are transferred to the aqueous phase by giving a force from the top to the bottom of the microtube by centrifugation (Figure 1). Although a large number of the droplets are broken when they pass through the interface, successful droplets form a lipid bilayer membrane, resulting in the generation of GVs. Usually, after centrifugation, white debris appears at the interface. This has to be removed entirely together with the oil phase. The formed GVs appear at the bottom of the tube because those are containing sucrose, which is heavier than glucose in the outer solution. A portion of the bottom fraction is collected carefully and transferred into a fresh tube for further experimentation. GVs can be washed with the same outer solution. Additional reagents can be supplied to the outer solution as needed while paying attention to the change in osmotic pressure. For example, ATP can be supplied to the exterior of the GVs at the same concentration as in the internal cell-free system, considering the possible leakage of ATP from the inside.

### The Storable Artificial Cell Mixture

To make the component solutions of artificial cells portable and easy to store, the mixed inner cell-free reaction solution (without DNA) and the outer solution were lyophilized. These are used to make a ready-to-use kit together with the dried lipid film and mineral oil. This enabled us to generate artificial cells within 30 min starting from the preparation of the lipid-oil to the formation of artificial cells. The successful protein synthesis using the lyophilized and rehydrated cell-free components has been reported previously (Hunt et al., 2017; Lee et al., 2020; Yang et al., 2021). Therefore, it is possible to ship the samples at ambient temperature while avoiding moisture. This has a great advantage for working outside the laboratory or exchanging materials between laboratories.

## Materials and Equipment

### Materials

• Glass tube: diameter, 10 mm; length, 50 mm (Maruemu Corp., cat. #0407-03)
• Microtube, 1.5 ml (Eppendorf, 3810X)
• PCR tube, 0.2 ml (Nippon Genetics, Co., Ltd., cat. # FG-021D)
• Parafilm (Bemis™, PM996)

### Reagents

• Chloroform (CHCl_3_) (Fujifilm, cat. #038-02606). CAUTION: the vapor and liquid forms of chloroform are toxic
• Mineral oil (MP Biomedicals, Inc., cat. #194836)
• 1-palmitoyl-2-oleoyl-glycero-3-phosphocholine (sodium salt), POPC (Avanti Polar, cat # 850457P)
• 1-palmitoyl-2-oleoyl-sn-glycero-3-phospho-(1′-rac-glycerol) (sodium salt), POPG (Avanti Polar, cat # 840457P)
• Sucrose (Fujifilm, cat. # 196-00015)
• d(+)-Glucose (Fujifilm, cat. # 049-31165)
• Cell-free system, e.g., PURE*frex*2.0 (GeneFrontier, cat. # PF201-0.25-5-EX)
• Genes of interest (under the control of the T7 promoter and ribosome-binding site)
• (Optional) Ficoll PM70 (Sigma-Aldrich, cat. #F2878)

### Reagent Preparation

• Phospholipids (e.g., POPC or POPG) are dissolved in CHCl_3_ at 100 mM in a glass vial and briefly processed with bath sonication.
• Sucrose and glucose solutions are prepared at 2 M with MilliQ water.
• Sol. I (Buffer) of PURE*frex*2.0 is preheated at 37°C for 5 min before mixing and placed on ice until use, as per the manufacturer’s guidelines.
• Sol. II (enzymes) and III (ribosome) of PURE*frex*2.0 are placed on ice until use, as per the manufacturer’s guidelines.
• Genes of interest are used as plasmids or linear DNAs.

### Equipment

• Aluminum block heating bath (e.g., TITEC Corp., cat. #DTU-2BN)
• Vortex (e.g., Scientific Industries, Inc., cat. #SI-T236)
• Nitrogen (N_2_) gas
• Centrifuge (e.g., TOMY Digital Biology, cat. #MX-307)
• Ultrasonic bath (e.g., Elma Schmidbauer GmbH, cat #S15H)
• Inverted fluorescence microscope (e.g., Olympus Life Science IX73) or confocal microscopy system (e.g., Nikon AIR)

**Table:** 

STEPWISE PROCEDURES		
**1. Inner solution preparation (5 min)**		
1.1	Prepare 20 µL of the inner solution containing 200 mM sucrose (e.g., a reaction mixture of a cell-free system containing the genes of interest and sucrose) (Figure 2.1)	When using Ficoll PM70, mix the prepared inner solution with 12% (w/v) Ficoll PM70 at this step
1.2	Keep on ice until the lipid-oil is ready	Keep at room temperature (r.t.) if the sample should avoid low temperature
**2. Outer solution preparation (2 min)**		
2.1	Prepare 500 µL of the outer solution containing 200 mM glucose in a 1.5 ml microtube	The prepared outer solution can be stored in a −20°C freezer for at least 1 month
2.2	Keep on ice	Keep at r.t. if the internal solution is r.t
**3. Lipid film preparation (5 min)**		
3.1	Transfer 100 µL of 100 mM phospholipid solution into a glass tube which was washed with pure CHCl_3_ in advance (e.g., for making POPC: POPG (70:30 mol%) membrane, mix 70 µL of 100 mM POPC and 30 µL of 100 mM POPG)	An inactive gas such as N_2_ or argon should be filled in the bottle of lipid powder or solution before restoring in a freezer
		**CAUTION**: CHCl_3_ is toxic for the respiratory tract and skin. Work within a hood wearing gloves and glasses
3.2	Dry up the solvent by gently flowing nitrogen gas from the top of the tube with vortex (Figure 2.2)	Dried lipids should be used immediately in the next step because it absorbs moisture easily
		**CAUTION**: The vapor of CHCl_3_ is toxic for the respiratory tract and eyes. Work within a hood wearing gloves and glasses
3.3	(Option) Completely dry up the solvent and remove the moisture under low pressure in a desiccator for overnight (or more) while avoiding light	If a fluorescent-labeled lipid is used, the glass tubes of the lipid film should be covered with aluminum foil to avoid light while drying up
**4. Lipid-oil preparation (8 min)**		
4.1	Add 500 µL of mineral oil to the lipid film and vortex vigorously for 20 s (Figure 2.3)	
4.2	Heat at 70°C for 1 min, then vortex for 30 s immediately (Figure 2.4)	**CAUTION:** Beware of burns when using an incubator with a hot temperature
4.3	Heat at 70°C for 1 min again, then vortex immediately until the oil has cooled down to room temperature (Figure 2.5)	If the solution becomes turbid, repeat heating again
**5. GV formation (8 min)**		
5.1	Add 20 µL of the prepared inner solution to the lipid-oil	
5.2	Vortex for 30 s until W/O droplets are formed homogeneously (Figure 2.6)	The W/O droplets should be formed until they are fully emulsified
5.3	Transfer all W/O droplets onto the outer solution of step 2.1	
5.4	Centrifuge at 10,000 × *g* for 5 min at 4°C	When the sample need to avoid low temperature, centrifuge at a moderate temperature (15–25°C)
**6. GV collection (2 min)**		
6.1	After the centrifugation, remove the upper oil layer and the debris completely by pipetting (Figure 2.7)	Chang the pipette tips frequently while removing the oil and debris. Residual oil may break the formed GVs during the collection from the bottom of the tube
6.2	Dip a fresh tip down to the bottom of the tube and slowly collect 20–40 µL of the GVs pellet (Figure 2.8)	
6.3	Transfer into a new tube	
6.4	Observe the formation of GVs by microscopy (Figure 2.9)	1 or 2 µL of the sample is enough for the microscopy observation
**7. Washing the GVs (8 min) (if necessary)**		
7.1	Add 300–500 µL of the outer solution to the collected GVs and mix well	
7.2	Centrifuge at 10,000 × *g* for 5 min at 4°C	When the sample need to avoid low temperature, centrifuge at a moderate temperature (15–25°C)
7.3	Dip a fresh tip down to the bottom of the tube and slowly collect 20–40 µL of the solution	
7.4	Transfer into a new tube	

**Table:** 

Troubleshooting	
Problem	Solution
(Step 1.1) A small amount of Ficoll PM70 cannot be measured accurately	Measure 240 mg of Ficoll PM70 powder and dissolve in 10 ml of Milli Q water, then aliquot 100 μL into PCR tubes. After lyophilization, store in a vacuum desiccator with low pressure. The resulting tubes containing 2.4 mg of Ficoll PM70 can be used with 20 μL of the inner solution
(Step 3.1) The lipids does not completely dissolve in CHCl_3_ and looks turbid	Reduce the concentration of the lipid solution (e.g., 25 mM). Do not change the total amount of lipids used to prepare the lipid-oil solution
(Step 4.2 and 4.3) The lipid film does not dissolve well in oil	Raise the heating temperature or/and extend the heating time (e.g., 70–90°C for 1–5 min)
(Step 4.3) The lipid-oil solution became turbid after cooling down	An excessive cooling may result in insolubility of the lipids. Repeat step 4.3
(Step 6.2) Lipid debris appeared over the precipitated GVs layer	Remove the debris by gently pipetting or take only GVs avoiding the debris
(Step 6.4) (a) A large amount of lipid or oil debris appeared on the formed GV membrane	Moisture absorbed in the lipid-oil may reduce the quality of the GVs formed. The lipid films should be used immediately after their preparation (step 3.2), or should be stored in a vacuum desiccator until just before use (step 3.3). Additionally, mineral oil should be stored under low pressure at least overnight just before use (step 4.1)
(Step 6.4) (b) No GVs were observed or very few	Ensure that the osmotic pressure of the outer solution is equal to that of the inner solution. When an osmometer is not available, adjust the osmolarity of the outer solution equivalent to the inner one, by repeating the increase of glucose concentration by 100 mM
GVs are not stable and break within 1 h after the preparation	Adjust the osmotic pressure of the outer solution. See troubleshooting for step 6.4 (b)

### Anticipated Results

**FIGURE 2:**
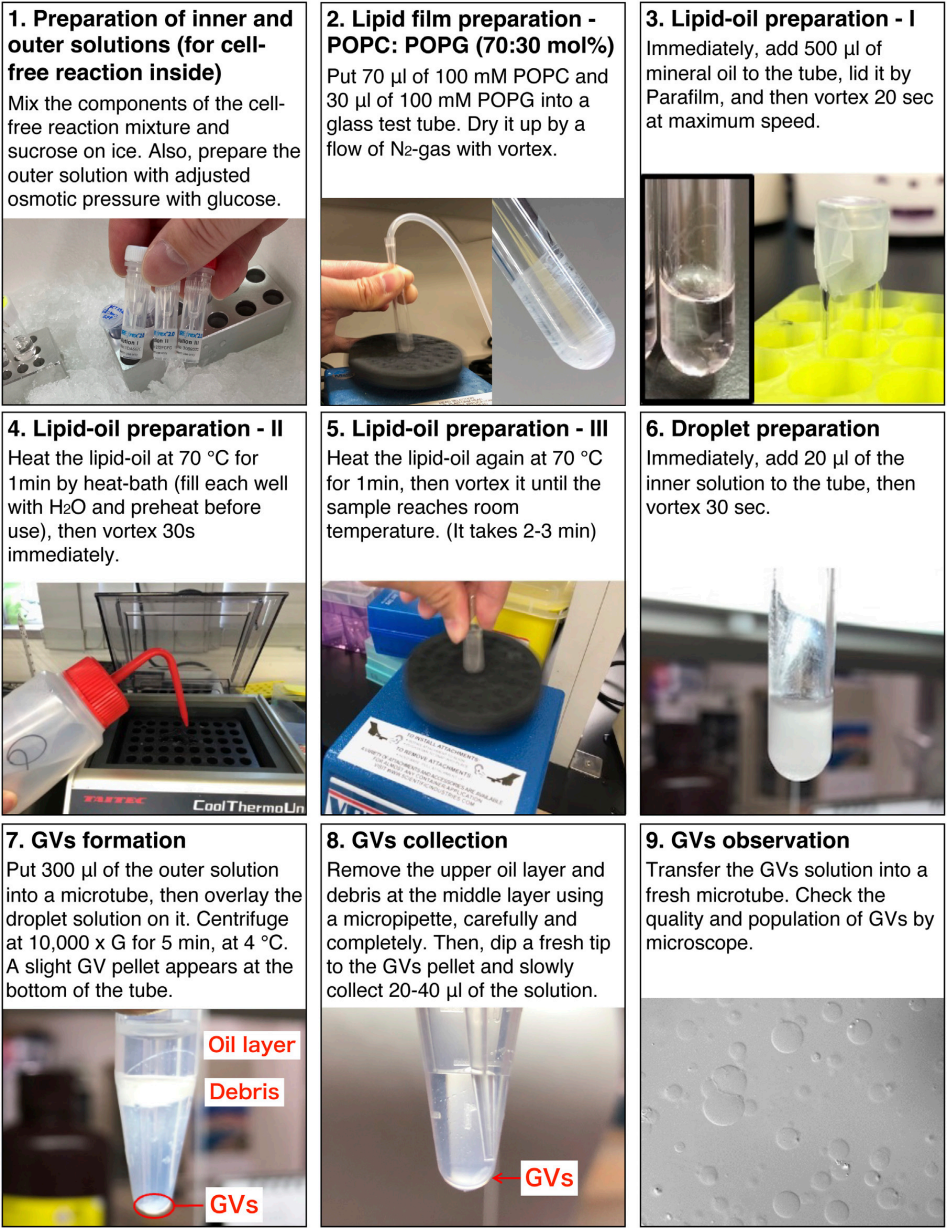
Stepwise protocol for the preparation of artificial cells. The details of the inner and outer solutions are provided in Tables 2, 3, respectively.

**FIGURE 3:**
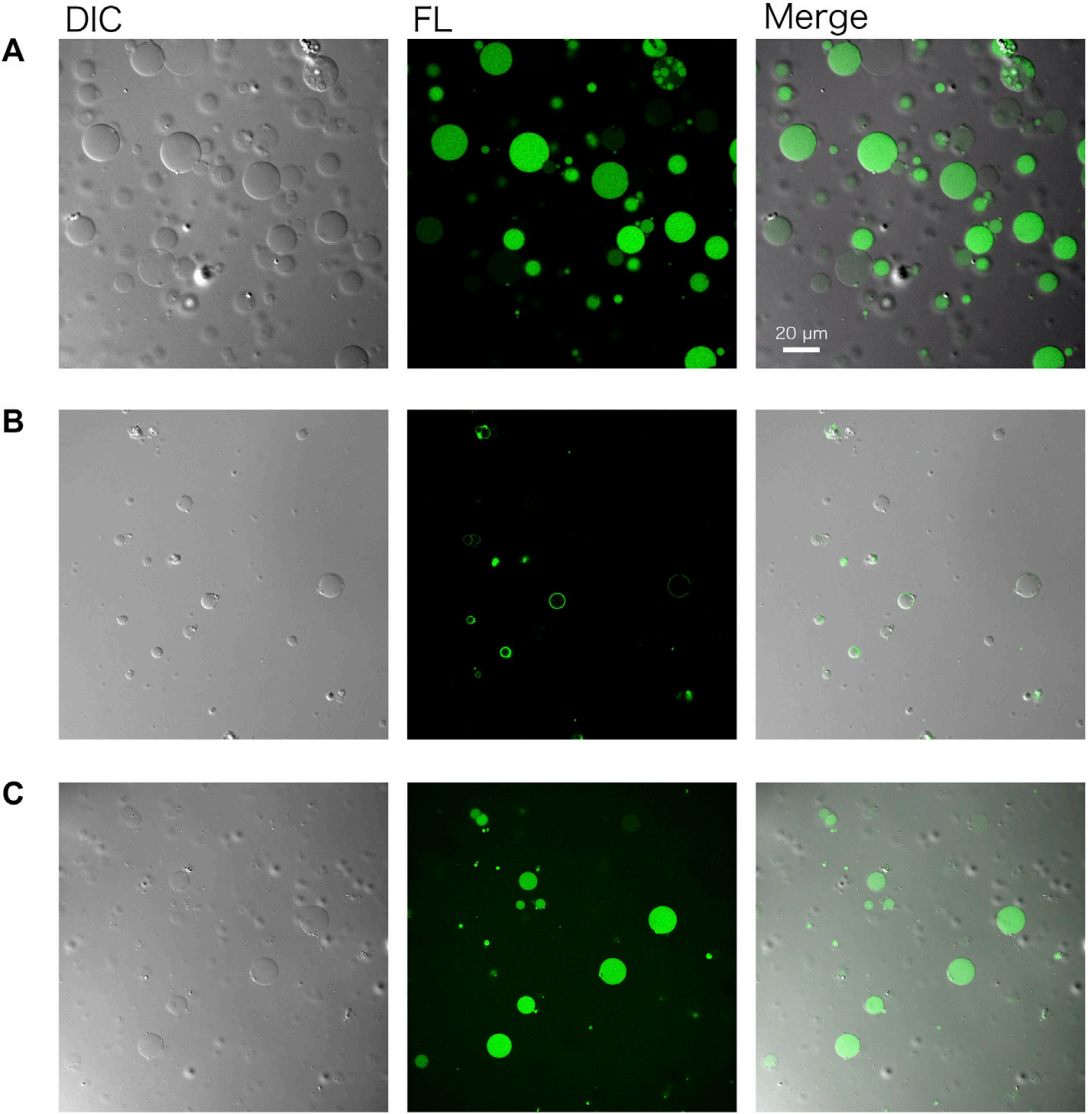
Confocal microscopy images of artificial cells. **(A)** Green fluorescent protein (GFP) and **(B)** a fusion protein of PlsY-GFP were synthesized in GVs. **(C)** GFP was synthesized using the lyophilized and rehydrated PURE system inside GVs. In any case, the GV membrane was composed of POPC: POPG (50:50 mol%). Images were obtained using a Nikon confocal microscopy A1R system. DIC: differential interference contrast, FL: fluorescence image.

GVs encapsulating a cell-free system (PURE system) and DNA of *gfp* were prepared by following the protocol described herein (Figure 2), using the outer solution described in Table 3. The resulting GVs were incubated at 37°C for 1–3 h to perform protein synthesis inside. Before incubation, 20 ng/μL of RNaseA were added to the exterior of the GVs to eliminate possible protein synthesis outside the GVs. ATP was also added to the outside of the GVs to prevent the leakage of the encapsulated ATP. The reacted GVs were observed by a confocal microscope equipped with a differential interference contrast unit. Using a 488 nm laser, we observed the synthesized green fluorescent protein (GFP) within the GVs consist of 50 mol% POPC and 50 mol% POPG (Figure 3A **)**.

A membrane protein was also synthesized using the same method by simply replacing the DNA. PlsY (glycerol-3-phosphate acyltransferase) is an integral membrane protein and was synthesized inside GVs. The gene encoding PlsY was fused to the GFP gene at the C terminus of the *plsY* DNA to visualize the product by microscopy. Note that modification of the N terminus of membrane proteins should be avoided because it may affect the co-translational spontaneous membrane localization of the proteins. Figure 3B shows successful protein synthesis in GVs and their localization onto the GV membrane.

Protein synthesis inside GVs was again performed using the lyophilized and rehydrated PURE system. All of the components of the reaction mixture including sucrose were mixed and lyophilized. After the rehydration of the PURE system, DNA encoding GFP was added and encapsulated inside GVs. The observed result was the same as that observed using the normal PURE system (Figure 3C).

## Discussion

Although the construction of artificial cells will provide a deepened understanding of the life system of cells, technical restrictions are preventing the development of this research field. Many protocols for GV formation have been published to date, but, in many cases, those are optimized within individual laboratories in detail and often contain unpublished tips and knowledge. This fact sometimes impedes the reproducibility of the results when other researchers work for GV formation by following the method reported in the article.

**FIGURE 4:**
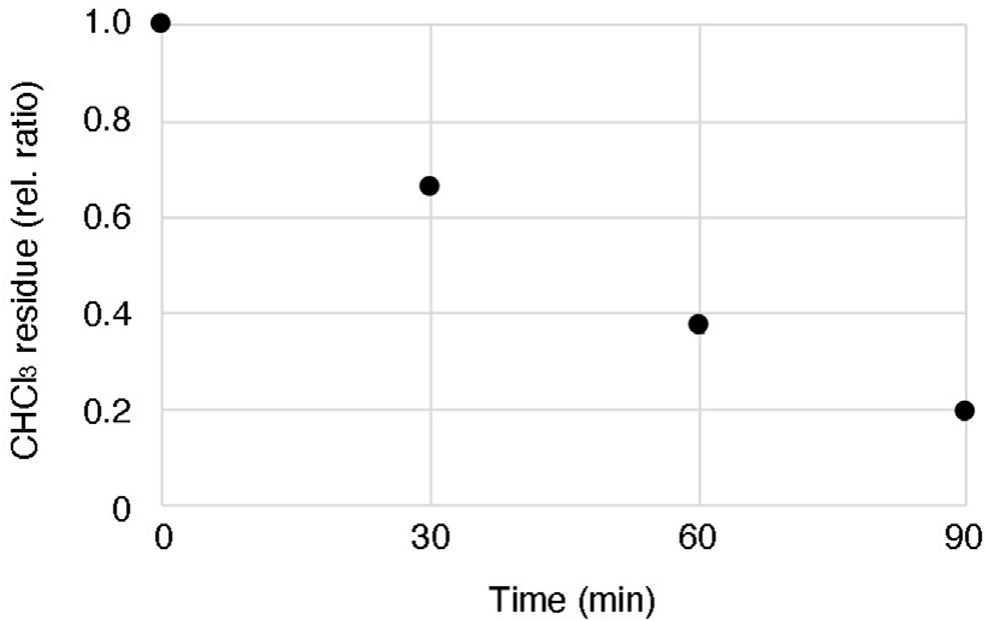
Residual chloroform in oil after heating and stirring. 200 µL chloroform containing 100 mM lipid (POPC 50 mol%: POPG 50 mol%) was added into 1 ml mineral oil, and then the lipid-oil mixture was heated at 60°C with stirring. The weight of the mixture was measured every 30 min until 90 min. The data were based on three independent replicates of the experiments.

According to one of the standard methods, a mixture of lipid and oil is prepared by adding the lipids dissolved in chloroform directly to the mineral oil and evaporating the chloroform by heating and stirring (Fujii et al., 2014; Uyeda et al., 2021). However, in this method, we found that a certain amount of chloroform remains in the oil solution even after evaporating the solvent for 90 min at 60°C with stirring (Figure 4). Although the effect of the residual solvent on the cell-free system might be negligible, there is a possibility that the reproducibility of the obtained GV quality may not be stable depending on the degree of residual solvent. Our proposed method can circumvent this risk, as the solvent is evaporated before mixing with the oil. As the other possibility, moisture in the oil or oxidation of the lipids may reduce the quality of the formation of GVs, as suggested by Robinson’s group (Moga et al., 2019) and Dekker’s group (Van de Cauter et al., 2021). To avoid these problems, the dried lipid film should be stored in a desiccator with low pressure under a stable temperature.

The use of lyophilized cell-free mixture and dried lipid films not only enables further reduction of the preparation time but also has the potential to expand the versatility of artificial cell technology. This is extremely useful for working outside the laboratory and for shipping samples. Moreover, it may be applied as a ready-to-use biosensor kit ​for the detection of DNA sequences derived from specific viruses without the use of special equipment. For example, by combining with a signal amplification system (Sato et al., 2019), the presence of pathogenic viruses in an environment or biological samples can be detected outside the laboratory. We believe our method will be the basis for the development of artificial cell engineering.

## Data Availability

The raw data supporting the conclusions of this article will be made available by the authors, without undue reservation.
